# Etiology and clinical profile of children and adolescents with disorders of sex development (DSD) presenting with ambiguous external genitalia

**DOI:** 10.1186/1687-9856-2013-S1-P195

**Published:** 2013-10-03

**Authors:** Sachin Mittal, Premlata Varthakavi, Manoj Chadha, Nikhil Bhagwat, Tejal Lathia, Ameya Joshi, Pratibha Pawal, Bharat Sharma

**Affiliations:** 1 T.N. Medical College & B.Y.L. Nair Hospital, Mumbai, India

## Aims

The international consensus statement on management of DSD, based on karyotype,has been clinicallyaccepted.Our aim was to study the clinical profile and etiology in patients with ambiguous external genitalia,using the new DSDclassification.

## Methods

We retrospectively assessed the records of patients, presenting with genital ambiguity, between 2009-2012,to the endocrine clinic of a tertiary care municipal hospital. The patients were classified on the basis of clinical features, hormonal investigations, imaging studies, karyotype and laparoscopy/biopsy, as indicated.

## Results

42 patients (age-neonate to 18 years, 14 (46 XX DSD), 26(46XY DSD) and 2(sex chromosome DSD) were evaluated.46 XX DSD was due to Congenital Adrenal Hyperplasia (CAH) (12/14) and SyndromicDSD(2/14).All presented with clitoromegaly and labioscrotal fusion. 5/12presented in infancy, with Adrenal crisis and severe (prader stage ≥3) virilization(Salt Wasting CAH), 7 had Simple VirilizingCAH.

Hypospadias was the most common presentation in 46XY DSD. Partial Androgen Insensitivity syndrome (PAIS) (8/26, 30%) was the most common etiology.4 had 5 alpha reductase deficiency, (1 had isolated micropenis and1 cryptorchidism with PraderWilli Syndrome while other 2 had hypospadias). 4 patients had Complete Androgen insensitivity Syndrome, 2(Pure Gonadal dysgenesis), 1 (SyndromicDSD),1 (CAH, 21 hydroxylase deficiency with peripheral precocious puberty), 1 (Vanishing testis syndrome). 5 patients had inconclusive biochemical profile.

6 patients presented with virilization at puberty. Though gender identity prior to puberty was female, history suggestive of conflict regarding the gender role was present. 4/6 were reassigned a male gender, while 2 continued as females.

1 patient of Sex Chromosome DSD, had Ovotesticular DSD with rare mosaic karyotype of 46XX(p-)(P21-23)/45X 80%/20%, while other had 46XY/46XX 58%/42%chimerism.

## Conclusion

46XY DSD comprised 60% cases with genital ambiguity. PAIS is the most common etiology of 46XY DSD and CAH of 46XX DSD. Subjects presenting for the first time in the peripubertal period with virilization, pose a bigger challenge to the treating team, in terms of gender role, identity and sex reassignment, along with difficulties in acceptance in society.The limitation of the study was lack of genetic confirmation, especially in inconclusive cases.

